# The trypanosomatid (Kinetoplastida: Trypanosomatidae) parasites in bees: A review on their environmental circulation, impacts and implications

**DOI:** 10.1016/j.cris.2025.100106

**Published:** 2025-01-21

**Authors:** Rossella Tiritelli, Giovanni Cilia, Tamara Gómez-Moracho

**Affiliations:** a Research Centre for Agriculture and Environment (CREA-AA), Council for Agricultural Research and Agricultural Economics Analysis, Bologna, Italy; b Department of Parasitology, Biochemical and Molecular Parasitology Group CTS-183, University of Granada, Granada, Spain; c Institute of Biotechnology, University of Granada, Granada, Spain

**Keywords:** *Lotmaria passim*, *Crithidia bombi*, *Crithidia mellificae*, Molecular epidemiology, Interspecific transmission, Pathology

## Abstract

Trypanosomatids, obligate parasites capable of impacting insects' hindgut, have recently obtained considerable attention, especially about their effects on bees. While *Crithidia mellificae* and *C. bombi* were initially discovered and studied in honey bees and bumblebees, respectively, molecular techniques revealed *Lotmaria passim* as the predominant trypanosomatid in honey bees globally. New species like *C. expoeki* and *C. acanthocephali* have also been identified. These parasites have complex life cycles involving various host developmental stages and are transmitted horizontally within and outside colonies through direct contact, oral interactions, and contaminating flowers with infected faeces. The impact of trypanosomatids on honey bee colony health remains uncertain. In bumblebees, studies highlighted the widespread presence of *C. bombi*, affecting colony and individual fitness, development, and foraging behaviour. Bee trypanosomatids have been detected in various species, including other insects, and mammals, suggesting diverse epidemiological pathways and potential effects that warrant further investigation. Biotic factors, including co-infections, gut microbiota, food contamination, and abiotic factors like environmental conditions, pesticides, and urbanization, play crucial roles in infection dynamics. This review aimed to summarise key research on trypanosomatid transmission and infection in both managed and wild bees, focusing on the influence of biotic and abiotic factors. The work highlights significant gaps in current knowledge and provides a valuable foundation for future studies. Understanding the pathogenicity and infection dynamics of trypanosomatids, along with the impact of environmental factors, is essential for developing effective conservation strategies that support pollinator health and overall ecosystem resilience.

## Highlights

• Trypanosomatids are obligate parasites infecting the hindgut of insects, especially bees.
• *Crithidia mellificae* and *Lotmaria passim* are the main trypanosomatids of honey bees.
• *C. bombi,* discovered in bumblebees, can infect both managed and wild species.
• The transmission occurs during the foraging on flowers or by contaminated water.
• Biotic and abiotic factors increase the host susceptibility, promoting the infection.

## 1 Introduction

Trypanosomatids (Kinetoplastida: Trypanosomatidae) are unicellular obligate, flagellated parasites capable of infecting a wide range of organisms, from plants to invertebrates and vertebrates (Borghesan et al., 2013; Jaskowska et al., 2015; Stevens et al., 1999). Dixenous flagellates, such as *Trypanosoma* spp. and *Leishmania* spp. which are agents of infections in humans and domestic animals (Kaufer et al., 2017), require two hosts to complete their lifecycle: an insect as a vector, and a vertebrate or a plant as the final host (Frolov et al., 2021; Maslov et al., 2013). Conversely, monoxenous trypanosomatids (e.g. *Leptomonas* or *Crithidia*) complete their development in a single host, often infecting invertebrates and particularly insects (Maslov et al., 2013; Schwarz et al., 2015).

During their life cycle, trypanosomatids display different morphotypes which are based on the relative position of their flagellum and kinetoplast (mitochondrial DNA (Lukeš et al., 2002)) in relation to their nucleus (Hoare and Wallace, 1966). These features have been frequently used for taxonomical purposes. Within their hosts, trypanosomatids are quite pleomorphic and can transition from motile to sessile forms (Buendía-Abad et al., 2022a). They have adapted to different environments for which they have developed strategies such as the secretion of biofilm which confers them resilience against different stressors (Carreira de Paula et al., 2024).

Despite neglected, research on insect trypanosomatids has increased in recent decades due to their negative impact on pollinators (e.g. in bumblebees (Brown et al., 2003a), solitary bees (Strobl et al., 2019) and their potential association with negative effects observed in honey bees (*Apis mellifera* L. 1758) (Gómez-Moracho et al., 2020).

The first evidence of flagellates in bees dates back to the mid-to-late 20th century with *Crithidia mellificae* being described in honey bees (Langridge and McGhee, 1967; Schmid-Hempel and Tognazzo, 2010) and, shortly after, *Crithidia bombi* in bumble bees (Lipa and Triggiani, 1988).

*C. mellificae* was discovered for the first time in Australia in 1967 (Langridge and McGhee, 1967; Schmid-Hempel and Tognazzo, 2010), but for nearly 40 years, this parasite received little to no attention until an increase in its prevalence in honey bees was reported (Cepero et al., 2014; Cornman et al., 2012; Runckel et al., 2011). *C. mellificae* was believed to be the only trypanosomatid capable of infecting honey bee colonies. However, modern molecular genetic tools enabled the discovery of a new species, called *Lotmaria passim* (Cepero et al., 2014; Schwarz et al., 2015). In fact, it was subsequently confirmed that all molecular data obtained from previous studies corresponded to this new species (Schwarz et al., 2015). *L. passim* is currently considered the most prevalent trypanosomatid detected in honey bees worldwide (Buendía-Abad et al., 2018; Castelli et al., 2019; Palmer-Young et al., 2022; Ravoet et al., 2015; Schwarz et al., 2015; Stevanovic et al., 2016). Lately, *L. passim* has also been detected in bumblebees (Michalczyk and Sokół, 2022) and solitary bees (Strobl et al., 2019). More recently, another trypanosomatid, *Crithidia acanthocephali,* was reported for the first time in honey bee colonies in Spain (Bartolomé et al., 2022; Buendía-Abad et al., 2022a).

*C. bombi,* was found in bumble bees (*Bombus* Latreille, 1802) in 1988 (Lipa and Triggiani, 1988), in which it affects several bumblebees’ life traits (i.e. fecundity, longevity, or behaviour) (Schmid-Hempel et al., 2019). Since then, any trypanosomatid detected in bumblebees was considered as *C. bombi*. But, likewise to *C. mellificae*, sequence analysis on *C. bombi* isolates led to the discovery of *Crithidia expoeki* (Gerasimov et al., 2019; Schmid-Hempel and Tognazzo, 2010) commonly infecting bumble bees (Cohen et al., 2021; Graystock et al., 2020; Lim et al., 2023; Schmid-Hempel and Tognazzo, 2010; Tripodi et al., 2018). Recently, *C. bombi* was shown to infect solitary bees (Figueroa et al., 2021b; Tiritelli et al., 2024).

In recent years, there has been an increase in studies focusing on insect trypanosomatids. Understanding of the biology, ecology, and genetics of the trypanosomatid *C. bombi* in bumblebees has been well-established and studied for over thirty years, thus becoming a model system of host-parasite evolutionary ecology (Schmid-Hempel et al., 2018, 1999; Schmid-Hempel and Schmid-Hempel, 1993; Shykoff and Schmid-Hempel, 1991a). However, it was not until recently that research on trypanosomatids infecting honey bees intensified (Aguado-López et al., 2023; Buendía-Abad et al., 2022a, 2023). Although some trypanosomatids were believed to be specific to their original host, the latest studies have shown otherwise.

This review aimed to consolidate the most recent data and updates concerning the spread of trypanosomatids in bees and other hymenopterans, insects and mammals. Additionally, we intend to elucidate the influence of both biotic and abiotic factors on the transmission of these parasites.

## 2 Trypanosomatids infection and transmission in bees

**Fig. 1:**
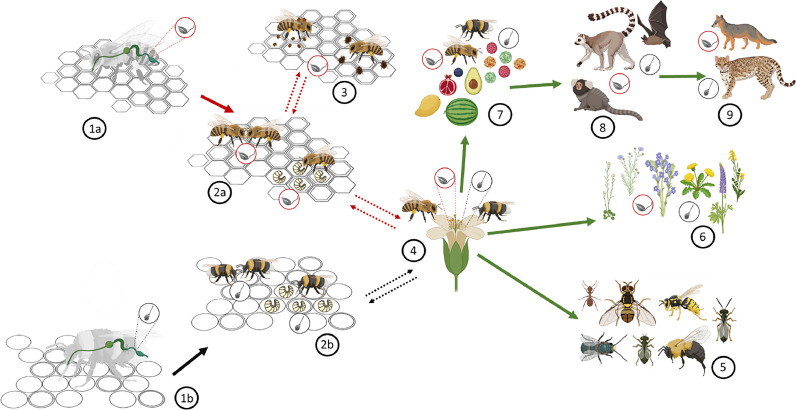
Tentative proposal of a transmission route of trypanosomatids in bees, *L. passim* (1a) and *C. bombi* (1b). Within colonies both parasites are directly transmitted among honey bees (2a) and bumblebees (2b) through grooming activities, contact with infected faeces, contaminated food resources and larval stage transmission via nurse bees. Whitin honey bee colonies, the parasites might also be shared with other parasites such as *V. destructor* or *A. tumida*, potentially serving as passive vectors (3). Both honey bees and bumblebees can contaminate floral resources (pollen and nectar) with their faeces and acquire the parasite by coming into contact with previously deposited contaminated faeces, thereby spreading the infection within colony populations (4). The parasite, originating from contaminated floral sources, can infect other pollinators or flower-visiting insects (5) and spread to various plant species (6). Besides, there is a potential transmission route to mammals through the food chain. Ingestion of contaminated bees, fruits, seeds, and pollen (7) could potentially transfer the parasite to bats and primates (8). Carnivores might acquire trypanosomatids by consuming previously infected prey or occasionally by consuming infected insects and fruits (9). Created with BioRender.com.

Bee infection with trypanosomatids occurs through fecal-oral transmission, involving the ingestion of infective parasite forms (Schmid-Hempel et al., 2019). The transmission can occur horizontally among bees both outside and inside the colony. Bees are usually exposed to the parasite outside the colony during their foraging activity, particularly when they share the same floral resources. These flowers become contaminated during bee visitation while depositing their faeces (Burnham et al., 2021; Cisarovsky and Schmid-Hempel, 2014; Durrer and Schmid-Hempel, 1994; Yañez et al., 2020), thus enabling horizontal transmission between colonies and posing a threat to other pollinators or even mammals (Fig. 1). Afterwards, the parasite will spread within the colony through direct contact between nestmates, contacts with contaminated materials, such as faeces (Gegear et al., 2005; Ruiz-González and Brown, 2006; Schmid-Hempel, 2001), or infected larvae (Folly et al., 2017). Trophallactic contact for food transfer could serve as a potential route of transmission for trypanosomatids in honey bees (Seeley, 1995), although this remains to be proven.

Vertical transmission has been described for *C. bombi* between bumblebee colonies. Thus, young infected queens that establish a new colony after hibernation will transmit the parasite to the next generation (Schmid-Hempel et al., 1999). The parasite infection persists in overwintering gynes, from which it can spread to future generations (Cisarovsky and Schmid-Hempel, 2014). The absence of infected eggs seems to exclude the possibility of vertical transmission within the colony in both honey bees (Arismendi et al., 2022) and bumblebees (Imhoof and Schmid-Hempel, 1999).

So far, there is no evidence that trypanosomatids can be vectored between bees. Nonetheless, recent molecular evidence of *C. bombi, L. passim* and *C. mellificae* in the small hive beetle *Aethina tumida* (Fernandez De Landa et al., 2020; Nanetti et al., 2021), and *L. passim* in the mite *Varroa destructor* (Quintana et al., 2021) suggests that these honey bee pests may serve as potential vectors.

Once trypanosomatids are ingested, they colonize the hindgut of bees where they attach to the host's intestinal epithelial cells (Lipa and Triggiani, 1988; Schwarz et al., 2015; Strobl et al., 2019). Species such as *L. passim* (Buendía-Abad et al., 2022a) and *C. acantocephali* (Buendía-Abad et al., 2022b), have been shown to remodel the flagellum into an “attachment plaque” and transition to the haptomonad form, allowing the parasite to remain fixed to the cell walls and potentially cause tissue damage (Buendía-Abad et al., 2022a, 2022b; Strobl et al., 2019). Once attached, the parasite rapidly multiplies (Popp and Lattorff, 2011; Schmid-Hempel and Schmid-Hempel, 1993), and infectious cells are consistently released in the bee's faeces. For instance, *C. bombi* is observed in faeces 3–4 days after infection and their numbers continue to rise steadily for an additional 10–12 days (Schmid-Hempel et al., 1999; Schmid-Hempel and Schmid-Hempel, 1993). In infected bumblebees, their faeces can contain a high concentration of parasite cells, with even a minimal count of 5000 cells being sufficient for parasite transmission (M.J.F. Brown et al., 2003b; Logan et al., 2005). This infection also enhanced the defecation rate of bumblebees on flowers, increasing parasite transmission efficiency (Figueroa et al., 2019).

The infection dynamics may depend on multiple factors, including transmission routes, infective dose, parasite strains, adaptation and virulence, host immunity system and environmental conditions (Schmid-Hempel, 2001). Studies in *C. bombi* indicate that even a few numbers of cells can infect bumblebees, although not all strains have the same infectivity. This infectivity depends on the parasite strain as well as on the genotype of the host (Schmid-Hempel and Ebert, 2003). For example, *C. bombi* cells vary in the intensity of infection they produce in different *Bombus terrestris* colonies, but they do not necessarily increase their infection success in related bumblebees after repeated transmissions (Yourth and Schmid-Hempel, 2006). Additionally, colony heterogeneity can play a crucial role in preventing acute parasite infection in bumblebees (Imhoof and Schmid-Hempel, 1998), or in reducing the transmission rate of *C. bombi* when heterogeneity is high (Shykoff and Schmid-Hempel, 1991a). On the other side, a short latency period between infection and the shedding of *C. bombi* cells has been shown to enhance the intensity of subsequent infections in other bumblebees (Schmid-Hempel and Schmid-Hempel, 1993).

## 3 Trypanosomatids in honey bees

In honey bees, the first trypanosomatid identified was *C. mellificae* in the late 60s (Langridge and McGhee, 1967), but nowadays other species such as *L. passim* (Schwarz et al., 2015), *C. acanthocephali* or *C. expoeki* (Bartolomé et al., 2020) have been confirmed.

**Fig. 2:**
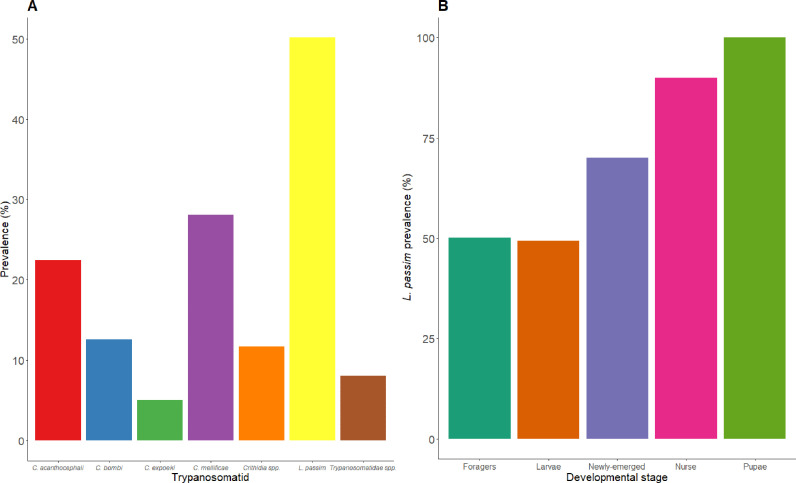
The average prevalence of trypanosomatids in honey bee colonies. A) Prevalence of trypanosomatids species in honey bee foragers. B) Prevalence of *L. passim* across honey bee developmental stages (foragers, larvae, newly emerged bees, nurse and pupae). Data from 54 studies were used to compile these figures. In cases where the studies did not identify the *Crithidia* or trypanosomatid at the species level, the terms “*Crithidia* spp.” or “'Trypanosomatidae spp.” were used, respectively, to represent these findings.

*L. passim* is the most widespread trypanosomatid in apiaries worldwide (Arismendi et al., 2016; Bartolomé et al., 2020; Ngor et al., 2020). For instance, in South America, its prevalence ranges from 13% to 72% (Castelli et al., 2019), while in North America it ranges from 14% to 25% (Lim et al., 2023; Xu et al., 2018). In Europe, *L. passim* prevalence varies from 13% to 80%, around 85% in New Zealand (Hall et al., 2021), 41% in Japan (Yamamoto et al., 2023) and 30% in India (Vavilova et al., 2017) (Fig. 2). Studies indicate that Africanized honey bees are susceptible to *L. passim* too (Castelli et al., 2019), with occurrences also reported in Asian populations of *Apis cerana* and *A. dorsata* (Vavilova et al., 2017)*. L. passim* has also been detected in honey samples from global trade markets (Ribani et al., 2020), with higher prevalence observed in samples from northern Italy (Ribani et al., 2021).

In contrast, *C. mellificae* has been sporadically identified in honey bees, with prevalence ranging from less than 1 % in Italy (Bordin et al., 2022), 2–28 % in U.S.A. (Lim et al., 2023; Ngor et al., 2020; Xu et al., 2018), 7–61 % in Spain (Bartolomé et al., 2020; Buendía-Abad et al., 2023) and 38% in the Czech Republic (Mráz et al., 2021). *C. bombi*, typically associated with bumblebees, has also occasionally been found in European honey bee colonies, with prevalence ranging from less than 1–54 % (Aguado-López et al., 2023; Bartolomé et al., 2020), and in the U.S.A., which ranges from less than 4 % (Cohen et al., 2021; Graystock et al., 2020). Other species like *C. acanthocephali* and *C. expoeki* have been detected in Spain (22%) (Bartolomé et al., 2020) and the U.S.A. (1–10 %), respectively (Cohen et al., 2021; Graystock et al., 2020).

Trypanosomatids have primarily been detected in honey bee foragers, but *L. passim* has also been found in larvae, pupae, and newly-emerged adults (Arismendi et al., 2022; Taric et al., 2020) (Fig. 2).

Within honey bee colonies, infection levels of *L. passim* seem to exhibit a seasonal pattern with slight variations among geographical regions. For instance, in the north hemisphere, *L. passim* is more prevalent during spring months in warmer regions like Spain or Serbia (Buendía-Abad et al., 2023; Vejnovic et al., 2018), although it seems to peak as well in the warmest autumn months (i.e. September; (Vejnovic et al., 2018)). Contrastingly, in the south hemisphere, the incidence of *L. passim* is more frequent during the autumn (i.e. New Zealand; (Hall et al., 2021)) and autumn-winter seasons (i.e. Chile; (Vargas et al., 2017)).

Experimental infections at the individual level have demonstrated reduced longevity in honey bees infected with *C. mellificae* or *L. passim* (Gómez-Moracho et al., 2020; Strobl et al., 2019). However, other studies on artificial infections found no significant reduction in survival time underscoring the need for further investigation to understand the pathogenicity of these parasites fully (Arismendi et al., 2020; Higes et al., 2016; Liu et al., 2020). Nonetheless, *L. passim* infection has been shown to negatively impact the nutritional status of infected honey bees as it decreases the stored vitellogenin in the fat body and reduces the transcription of nutritional-related genes (i.e. glycolysis) (Liu et al., 2020). Although no epithelial lesions have been observed in honey bees experimental infected with neither *C. mellificae* nor *L. passim* (Gómez-Moracho et al., 2020), alterations in metabolite absorption indicate potential negative effects on bee health (Buendía-Abad et al., 2022a, 2022b). Even if their pathogenicity remains uncertain, a positive correlation has been observed between *C. mellificae* infection and winter mortality (Ravoet et al., 2013).

## 4 Trypanosomatids in bumblebees

Since its discovery in 1980, *C. bombi* has been identified in numerous bumblebee species worldwide, with an average prevalence of 42 % in North America (Gegear et al., 2005; Lim et al., 2023; Tripodi et al., 2018), 39 % in South America (Arismendi et al., 2021; Plischuk et al., 2021), and 38 % in Europe (Goulson et al., 2012; Tiritelli et al., 2024). A total of 35 bumblebee species have been found susceptible to this parasite and other trypanosomatids (Table S1). Genetic diversification within host-parasite systems, such as *C. bombi, C. expoeki* and *Bombus* spp., can influence parasite prevalence. For instance, such prevalence peaks during high blossom periods and fluctuates seasonally along with variations in parasite genetics structure (Popp et al., 2012). On the other side, low genetic diversity in host populations leads to high parasite prevalence (Parsche and Lattorff, 2018; Whitehorn et al., 2011). Besides, bumblebees exhibited variation in the expression of immune-related genes in response to *C. bombi* infection, showing differences in immune competence among colonies (Schlüns et al., 2010).

*C. bombi* affects queens, males, and workers (Brown et al., 2003a, 2000; Plischuk et al., 2021; Votavová et al., 2022; Yourth et al., 2008), while larvae act as a source of contamination within the colony but do not exhibit signs of infection (Folly et al., 2017). At the colony level, *C. bombi* infection negatively impacts colony-founding success, size, and fitness (Brown et al., 2003a; Imhoof and Schmid-Hempel, 1999; Shykoff and Schmid-Hempel, 1991). At the individual level, infected queens show reduced male production, lower fitness and mass loss after stressful hibernation (Yourth et al., 2008). Conversely, *C. bombi* infection delayed ovarian development, and consequently oviposition, in worker bumblebees (Shykoff and Schmid-Hempel, 1991b), and a shift in resource reallocation toward the fat body (Brown et al., 2000). The parasite infection can reduce the competition with the queen and increase worker cooperation for a longer time (Shykoff and Schmid-Hempel, 1991b), even in some cases, *C. bombi* infection stimulated the egg development of workers correlated to the age and size (Korner and Schmid-Hempel, 2005).

*C. bombi* has been shown to impair the behaviour and cognition of infected bumblebees. For instance, they show an altered foraging behaviour with longer flights, visiting fewer flowers, and lower handling proficiency (Otterstatter et al., 2005), thus doubling the time and number of visits required to learn flower manipulation (Gegear et al., 2005). Besides, their visual learning is affected showing a lower ability to learn to discriminate flowers of different colours (Gegear et al., 2006). Together reducing foraging efficiency and potentially lowering colony fitness. Treatments like thymol and nicotine showed promise in reducing infection rates in commercial colonies although high concentrations of thymol led to significant mortality (Biller et al., 2015). Additionally, treatment with pulse light on *C. bombi* cultures reduced infection rates in artificially infected bumblebee workers, helping to mitigate colony-level damage (Naughton et al., 2017).

## 5 Trypanosomatids in other hymenopterans, insects and mammals

The spread of parasites among different species is facilitated through indirect transmissions, like sharing contaminated food resources (Burnham et al., 2021; Yañez et al., 2020), contaminated flowers (Graystock et al., 2015), contact with contaminated faeces (Manley et al., 2015; Piot et al., 2021; Yañez et al., 2012), mediated by vectors or other organisms (Gisder et al., 2009; Yañez et al., 2020), and through scavenging or predation (Genersch et al., 2010; Nanetti et al., 2021).

**Fig. 3:**
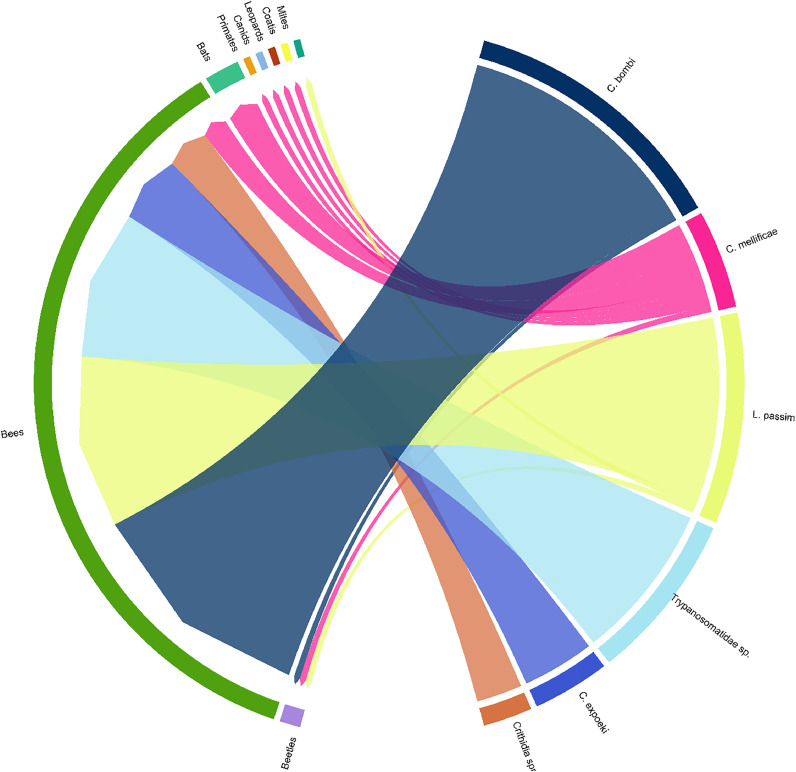
Detection of trypanosomatids in other arthropods (beetles, bees and mites) and mammals (bats, primates, canids, leopards and coatis). Number of findings is indicated by the width of the arrow.

Monitoring efforts have identified trypanosomatids in wild bees across different countries (Table S2 and Fig. 3) (Cilia et al., 2022; Cohen et al., 2021; Fernandez De Landa et al., 2023; Figueroa et al., 2021b, 2020; Lim et al., 2023; Ravoet et al., 2014; Tiritelli et al., 2024). *C. mellificae* infection has been identified in *Osmia cornuta, O. bicornis* and *Vespula squamosa* (Ravoet et al., 2014; Schwarz et al., 2015) (Fig. 3). Indeed, experimental infections of *C. mellificae* in *O. cornuta* have led to reduced survival of males and increased parasite spread through faecal-oral transmission (Strobl et al., 2019). Similar reductions in lifespan were observed in experimental infections of *C. mellificae* and *C. bombi* in *O. lignaria, Halictus ligatus* and *Megachile rotundata* (Figueroa et al., 2021a; Ngor et al., 2020). Among other pollinators, *C. bombi* has been detected in the faeces of artificially infected *Eristalis arbustorum* and *Eristalis tenax*, indicating that insects not typically hosting the parasite can still contribute to its transmission through shared food resources (Davis et al., 2021). *C. bombi, C. mellificae* and *L. passim* were evidenced in *A. tumida* , with *L. passim* also found in *V. destructor* (Fig. 3). However, no signs of infection have been observed in these arthorpods, nor has their ability to transmit these parasites been demonstrated.

While *C. mellificae* was believed to solely infect insects, recent studies in Brazil have isolated it from haemocultures of various mammals, including bats (*Anoura caudifer, Carollia perspicillata, Myotis lavali, Myotis izecksohni, Artibeus lituratus*), coatis (*Nasua nasua*), marmosets (*Callithrix* sp.), crab-eating foxes (*Cerdocyon thous*) and ocelot (*Leopardus pardalis*) (Dario et al., 2021; Rangel et al., 2019) (Fig. 3). Transmission likely occurs through contaminated fruit, infected insects, or predation (Dario et al., 2021) (Fig. 1). However, the extent of mammalian infection and its involvement in trypanosomatid transmission remains uncertain, necessitating further research to comprehend the epidemiological routes and effects of bee trypanosomatid infection on other vertebrates.

## 5 Biotic factors in trypanosomatids infections

Various biotic factors influence the occurrence and virulence of trypanosomatids among bees and their populations.

First of all, positive correlations have been found between trypanosomatids *L. passim* and *C. mellificae* with the microsporidium *Nosema ceranae* in honey bees colonies (Arismendi et al., 2020; Higes et al., 2016; Ravoet et al., 2013; Runckel et al., 2014, 2011; Schwarz and Evans, 2013; Tritschler et al., 2017; Vejnovic et al., 2018). Higher trypanosomatids virulence was found in bees co-infected with *N. ceranae*, causing an increase in mortality (Arismendi et al., 2020; Higes et al., 2016) and winter colony losses (Ravoet et al., 2013; Runckel et al., 2014)*.* The infection of *N. ceranae* in naturally *L. passim-*infected bees caused a drastic decrease in the expression of the antimicrobial peptides and vitellogenin, speeding up honey bee mortality (Arismendi et al., 2020). What is not clear is if trypanosomatid infections might occur as a secondary infection, resulting from a weakened immune defence after the microsporidia infection (Antúnez et al., 2009). Both microorganisms may synergistically increase lethality in infected bees.

The bee gut bacteria play a crucial factor in modulating host susceptibility to trypanosomatids infection (Fernandez De Landa et al., 2023; Koch and Schmid-Hempel, 2012; Palmer-Young et al., 2019; Schwarz et al., 2016). Alterations in gut bacteria have been associated with increased susceptibility to L. *passim* in honey bees, possibly due to dysbiosis, nutritional stress, or antibiotic application (Schwarz et al., 2016), even if this was not generally observed (Hubert et al., 2017; Liu et al., 2020). In bumblebees, the presence of gut bacteria rich in *Lactobacillus* Firm-5, *Apibacter* and *Gilliamella* spp. increased resistance to *C. bombi* infections (Koch and Schmid-Hempel, 2012; Mockler et al., 2018; Palmer-Young et al., 2019). A strong correlation was found between the gut microbiome composition of solitary bees *Xylocopa augusti, Eucera fervens* and *Lasioglossum* sp. with the presence and abundance of *C. bombi* (Fernandez De Landa et al., 2023).

Also, biological traits such as body size influence the risk of exposure to trypanosomatids (Cohen et al., 2021; Figueroa et al., 2021a; Malfi and Roulston, 2014; Tiritelli et al., 2024; Van Wyk et al., 2021). Larger bees have been associated with higher transmission rates of *C. bombi*, due to increased foraging and faeces production (Korner and Schmid-Hempel, 2005; Van Wyk et al., 2021), while smaller bees exhibited a higher infection of *Crithidia* spp. (Malfi and Roulston, 2014).

Flowers are a key factor in bee-parasite exposure and transmission as they act as a reservoir of contaminated faeces released by infected bees. Floral density may affect parasite transmission by, for instance, reducing the likelihood of bees encountering other infected individuals when floral availability is high (Piot et al., 2021). However, wild bee abundance in mass-flowering crops was associated with higher trypanosomatids prevalence in areas with a low non-crop flower abundance (Cohen et al., 2021). The higher prevalence and abundance of parasites in *B. pascuorum* were also related to higher floral abundance (Tommasi et al., 2023). In addition, floral shape has been shown to affect parasite transmission. Wider flowers are more prone to accumulate more faecal spots which become more accessible to bumblebees, especially, in shorter flowers which are less exposed to stressors such as UV light which might affect parasite survival (Pinilla-Gallego et al., 2022).

Poor nutritional status associated with *L. passim* infection caused a decrease in stored vitellogenin in the fat body of honey bees infected, as well as observed in bumblebees infected with *C. bombi* (Amdam et al., 2012; Brown et al., 2003b; Liu et al., 2020; Shykoff and Schmid-Hempel, 1991). Although no epithelial lesions have been observed in honey bees experimental infected with *C. mellificae* and *L. passim* (Gómez-Moracho et al., 2020), alterations in metabolite absorption indicate potential negative effects on bee health (Buendía-Abad et al., 2022a, 2022b).

The nutrition status of bees modulates parasite infections. Under nutritional stress, *Crithidia*-infected bumblebee workers exhibited a 50% increase in mortality (Brown et al., 2003a; Schmid-Hempel, 2001). Pollen availability influenced infection rates, with low nutrition leading to reduced parasite counts (Conroy et al., 2016), even if the administration of syrup with a high sugar concentration reduced the environmental persistence of *C. bombi* (Cisarovsky and Schmid-Hempel, 2014; Folly et al., 2020). The availability of nutrition resources can either amplify or dilute trypanosomatid virulence by affecting the aggregation of susceptible hosts and contaminated flowers (Civitello et al., 2015; Doublet et al., 2022; Westphal et al., 2003). Feeding *B. impatiens* with pollen from various *Helianthus* spp. and *Solidago* spp. significantly reduced *C. bombi* levels (Koch et al., 2019; LoCascio et al., 2019; Malfi et al., 2023), while *B. terrestris* fed with sunflower or heather pollen exhibited decreased fitness but increased resistance to the parasite (Vanderplanck et al., 2023). Pollen collected from *Osmia* during mass-flowering crops showed a lower prevalence of *Crithidia* spp. compared to post-mass flowering collections (Piot et al., 2021).

## 6 Abiotic factors in trypanosomatid infections

To facilitate the horizontal transmission of trypanosomatids through flower sharing, the parasite's viability on the flower surface is crucial (Figueroa et al., 2019). Factors such as UV radiation, temperature, and desiccation affect the survival of *C. bombi* on flowers (Figueroa et al., 2019; Schmid-Hempel et al., 1999), although *C. mellificae* exhibits high tolerance to elevated temperatures (Dario et al., 2021). Recent studies have shown *C. bombi* resilience to a wide temperature range, remaining non-infectious to bees only at extreme temperatures (Wolmuth-Gordon and Brown, 2023).

Among abiotic factors, the interaction between pesticides and parasites is notable (Harwood and Dolezal, 2020; Straub et al., 2022). Neonicotinoid exposure combined with *C. bombi* infection adversely affects queen survival and colony success in bumblebees (Fauser-Misslin et al., 2014). Different pesticides, like Amistad or imidacloprid, in combination with trypanosomatids, have resulted in weight loss, high mortality and immune response alteration in bumblebees (Askri et al., 2023), or increased parasite prevalence and abundance in honey bees, respectively (Erban et al., 2023). Experimental *C. bombi* infections on *B. terrestris* combined with exposure to high doses of glyphosate showed no impact on the gut microbiome of the bumblebees (Straw et al., 2023). However, the effects of these co-exposures on bees require further investigation.

Urbanization and anthropization can impact pollinator communities and parasite dynamics (Youngsteadt et al., 2015). While urban areas may support higher pollinator densities (McFrederick and LeBuhn, 2006; Osborne et al., 2008), this may also lead to increased parasite abundance. Studies suggest a higher prevalence of *C. bombi* in urban areas (Goulson et al., 2012; Theodorou et al., 2016), possibly due to bee aggregation. However, the presence of green habitats in fragmented landscapes might decrease the infection likelihood in certain bee species (Tommasi et al., 2023). Further research is needed to understand trypanosomatid transmission dynamics in urban settings.

## 7 Conclusions

Research on trypanosomatids has expanded knowledge about their distribution, prevalence, and impact on hosts, particularly honey bees and bumblebees. Species like *L. passim* and *C. bombi* have severe effects on bee colony health and individual fitness, spreading through complex interactions among bees, contaminated food resources, and the environment. The discovery of trypanosomatids in other insects and even mammals suggests broader ecological implications and potential new transmission routes. Future research should focus on transmission pathways, host-parasite interactions, and the influence of biotic (e.g., co-infections) and abiotic (e.g., pesticides) factors. A One-Health approach is essential to mitigate these parasites' impact on pollinators and ecosystems.

## Author contributions

All authors equally contributed to this work.

## Declaration of competing interest

The authors declare that they have no known competing financial interests or personal relationships that could have appeared to influence the work reported in this paper.

## Data Availability

Data will be made available on request.

## Appendix Supplementary materials

Image, application 1

Image, application 2
