# Supplementary material for: The trypanosomatid (Kinetoplastida: Trypanosomatidae) parasites in bees: A review on their environmental circulation, impacts and implications
Source: Curr Res Insect Sci. 2025 Jan 21;7:100106. doi: 10.1016/j.cris.2025.100106 (PMC11803887; doi:10.1016/j.cris.2025.100106)
Supplement: Supplementary file 1 [file mmc1.docx]

**Table S1.** Detection of bumblebee species at various stages infected with trypanosomatids reported in different countries.

| **Species** | **Stage** | **Country** | **Trypanosomatids** | **Reference** |
| --- | --- | --- | --- | --- |
| *Bombus* spp. | Workers | CL | *C. bombi* | (Barribeau and Schmid-Hempel, 2013) |
|  |  | GB |  | (Goulson et al., 2012) |
|  |  | SI |  | (Ocepek et al., 2021) |
|  |  | US | *C. bombi, C. expoeki,*  *C. mellificae,*  *L. passim* | (Tripodi et al., 2018) |
|  |  | US | Trypanosomatidae sp. | (Figueroa et al., 2021) |
| *B. lucorum/*  *B. cryptarum/*  *B. terrestris* | Workers | CH | *C. bombi* | (Korner and Schmid-Hempel, 2005) |
| *B. asiaticus* | Workers | IN | *L. passim* | (Vavilova et al., 2017) |
| *B. auricomus* | Workers | US | *C. bombi* | (Lim et al., 2023) |
|  |  |  | *Crithidia* spp. | (Malfi and Roulston, 2014) |
| *B. bifarius* | Workers | US | *Crithidia* spp. | (Cordes et al., 2012) |
| *B. bimaculatus* | Workers | US | *C. bombi* | (Graystock et al., 2020; Lim et al., 2023) |
|  |  |  | *C. expoeki* | (Graystock et al., 2020) |
|  |  |  | *Crithidia* spp. | (Cordes et al., 2012; Malfi and Roulston, 2014) |
|  |  |  | Trypanosomatidae sp. | (Averill et al., 2021; Figueroa et al., 2020) |
| *B. californicus* | Workers | US | *C. expoeki*,  *Crithida* spp. | (Cohen et al., 2021) |
| *B. centralis* | Workers | US | *Crithidia* spp. | (Cordes et al., 2012) |
| *B. dahlbomii* | Workers | CL | *C. bombi, L. passim* | (Arismendi et al., 2021) |
| *B. fervidus* | Workers | US | *C. bombi* | (Lim et al., 2023) |
|  |  |  | *Crithidia* spp. | (Cordes et al., 2012; Malfi and Roulston, 2014) |
| *B. flavifrons* | Workers | US | *Crithidia* spp. | (Cordes et al., 2012) |
| *B. frigidus* | Workers | US | *Crithidia* spp. | (Cordes et al., 2012) |
| *B. funebris* | Workers | PE | *C. bombi, L. passim* | (Plischuk et al., 2021) |
| *B. griseocollis* | Workers | US | *C. bombi, C. expoeki* | (Graystock et al., 2020; Lim et al., 2023) |
|  |  |  | *Crithidia* spp. | (Malfi and Roulston, 2014) |
|  |  |  | Trypanosomatidae sp. | (Averill et al., 2021; Figueroa et al., 2020; Lim et al., 2023) |
| *B. hortorum* | Workers | DE | *C. bombi* | (Erler et al., 2012) |
|  |  | IT | *C. bombi* | (Tiritelli et al., 2024) |
|  |  | CH | *C. bombi* | (Korner and Schmid-Hempel, 2005; Shykoff and Schmid-Hempel, 1991) |
| *B. huntii* | Workers | US | *Crithidia* spp. | (Cordes et al., 2012) |
| *B. hypnorum* | Workers | CH | *C. bombi* | (Shykoff and Schmid-Hempel, 1991) |
| *B. impatiens* | Workers | CA | *C. bombi* | (Gegear et al., 2005; Graystock et al., 2020; Lim et al., 2023) |
|  | Workers* | US | *C. bombi* | (Ngor et al., 2020; Pinilla-Gallego et al., 2020) |
|  | Workers | US | *C. expoeki* | (Graystock et al., 2020; Lim et al., 2023) |
|  | Workers | US | *Crithidia* spp. | (Averill et al., 2021; Malfi et al., 2023; Malfi and Roulston, 2014) |
|  | Workers | US | Trypanosomatidae sp. | (Averill et al., 2021; Figueroa et al., 2020) |
| *B. jonellus* | Workers | CH | *C. bombi* | (Korner and Schmid-Hempel, 2005) |
| *B. lapidarius* | Workers | CZ | *C. bombi* | (Votavová et al., 2022) |
|  | Workers | DE | *C. bombi* | (Erler et al., 2012; Popp et al., 2012) |
|  | Workers | IT | *C. bombi* | (Cilia et al., 2022) |
|  | Workers | CH | *C. bombi* | (Korner and Schmid-Hempel, 2005; Shykoff and Schmid-Hempel, 1991) |
|  | Workers | DE | *Crithidia* spp. | (Theodorou et al., 2016) |
| *B. lucorum* | Workers | IT | *C. bombi* | (Cilia et al., 2022) |
| *B. melanopygus* | Workers | US | *Crithidia* spp. | (Cordes et al., 2012) |
| *B. mendax* | Workers | CH | *C. bombi* | (Korner and Schmid-Hempel, 2005) |
| *B. mesomelas* | Workers | CH | *C. bombi* | (Korner and Schmid-Hempel, 2005) |
| *B. mixtus* | Workers | US | *Crithidia* spp. | (Cordes et al., 2012) |
| *B. monticola* | Workers | CH | *C. bombi* | (Korner and Schmid-Hempel, 2005) |
| *B. mucidus* | Workers | CH | *C. bombi* | (Korner and Schmid-Hempel, 2005) |
| *B. occidentalis* | Workers | US | *Crithidia* spp. | (Cordes et al., 2012) |
| *B. opifex* | Workers | PE | *C. bombi, L. passim* | (Plischuk et al., 2021) |
| *B. pascuorum* | Workers | DE | *C. bombi* | (Erler et al., 2012) |
|  | Workers | IT | *C. bombi* | (Cilia et al., 2022) |
|  | Workers | CH | *C. bombi* | (Shykoff and Schmid-Hempel, 1991) |
|  | Workers | DE | *Crithidia* spp. | (Theodorou et al., 2016) |
|  | Workers | IT | *Crithidia* spp. | (Tommasi et al., 2023) |
|  | Workers | PL | *L. passim* | (Michalczyk and Sokół, 2022) |
| *B. pauloensis* | Workers | BO | *C. bombi, L. passim* | (Plischuk et al., 2021) |
| *B. pensylvanicus* | Queens | US | *C. bombi* | (Lim et al., 2023; Tripodi et al., 2014) |
|  | Workers | US | *Crithidia* spp. | (Cordes et al., 2012) |
| *B. perplexus* | Workers | US | *C. bombi* | (Lim et al., 2023) |
|  | Workers | US | *Crithidia* spp. | (Malfi and Roulston, 2014) |
|  | Workers | US | Trypanosomatidae sp. | (Averill et al., 2021) |
| *B. pratorum* | Workers | IT | *C. bombi* | (Cilia et al., 2022) |
|  | Workers | CH | *C. bombi* | (Korner and Schmid-Hempel, 2005; Shykoff and Schmid-Hempel, 1991) |
| *B. vestalis* | Workers | DE | *C. bombi* | (Erler et al., 2012) |
| *B. pyrenaeus* | Workers | CH | *C. bombi* | (Korner and Schmid-Hempel, 2005) |
| *B. ruderarius* | Workers | CH | *C. bombi* | (Korner and Schmid-Hempel, 2005) |
| *B. ruderatus* | Workers | CL | *C. bombi* | (Arismendi et al., 2021; Cilia et al., 2022) |
|  | Workers | CL | *L. passim* | (Arismendi et al., 2021) |
| *B. sandersoni* | Workers | US | *C. expoeki* | (Graystock et al., 2020) |
| *B. sichelii* | Workers | CH | *C. bombi* | (Korner and Schmid-Hempel, 2005) |
| *B. simillimus* | Workers | IN | *L. passim* | (Vavilova et al., 2017) |
| *B. soroeensis* | Workers | CH | *C. bombi* | (Korner and Schmid-Hempel, 2005) |
| *B. sylvarum* | Workers | CH | *C. bombi* | (Shykoff and Schmid-Hempel, 1991) |
| *B. sylvestris* | Workers | IT | *C. bombi* | (Cilia et al., 2022) |
| *B. sylvicola* | Workers | US | *Crithidia* spp. | (Cordes et al., 2012) |
| *B. ternarius* | Workers | US | *Crithidia* spp. | (Cordes et al., 2012) |
|  | Workers | US | Trypanosomatidae sp. | (Figueroa et al., 2020) |
| *B. terrestris* | Workers, Males, Queens | AR | *C. bombi* | (Plischuk et al., 2017; Plischuk and Lange, 2009) |
|  | Workers | CL | *C. bombi* | (Arismendi et al., 2021) |
|  | Workers | CZ | *C. bombi* | (Votavová et al., 2022) |
|  | Workers | DE | *C. bombi* | (Erler et al., 2012; Popp et al., 2012) |
|  | Workers | IT | *C. bombi* | (Cilia et al., 2022) |
|  | Queens and Workers | CH | *C. bombi* | (Brown et al., 2003; Schmid-Hempel and Schmid-Hempel, 1993; Shykoff and Schmid-Hempel, 1991) |
|  | Workers | DE | *Crithidia* spp. | (Theodorou et al., 2016) |
|  | Workers | IT | *Crithidia* spp. | (Tommasi et al., 2023) |
|  | Workers | CL | *L. passim* | (Arismendi et al., 2021) |
|  | Workers | IT | *L. passim* | (Tiritelli et al., 2024) |
| *B. terrestris dalmatinus* | Workers* | IE | *C. bombi* | (Ruiz-González and Brown, 2006) |
| *B. terrestris/*  *B. lucorum* | Workers | CH | *C. bombi* | (Shykoff and Schmid-Hempel, 1991) |
| *B. trifasciatus* | Workers | IN | *L. passim* | (Vavilova et al., 2017) |
| *B. vagans* | Workers | US | *C. bombi, C. expoeki* | (Figueroa et al., 2020) |
|  | Workers | US | Trypanosomatidae sp. | (Averill et al., 2021; Figueroa et al., 2020) |
| *B. vosnesenskii* | Workers | US | *C. expoeki* | (Cohen et al., 2021) |
|  | Workers | US | *Crithidia* spp. | (Cordes et al., 2012) |
| *B. wurflenii* | Workers | CH | *C. bombi* | (Korner and Schmid-Hempel, 2005) |

Note: *Artificial infection; AR: Argentina; BO: Bolivia; CA: Canada; CH: Switzerland; CL: Cile; CZ: Czech Republic; DE: Germany; GB: Great Britain; IE: Ireland; IN: India; IT: Italy; PE: Peru; PL: Poland; SI: Slovenia; US: USA.

**References**

Arismendi, N., Riveros, G., Zapata, N., Smagghe, G., González, C., Vargas, M., 2021. Occurrence of bee viruses and pathogens associated with emerging infectious diseases in native and non-native bumble bees in southern Chile. Biol. Invasions 23, 1175–1189. https://doi.org/10.1007/s10530-020-02428-w

Averill, A.L., Couto, A. V., Andersen, J.C., Elkinton, J.S., 2021. Parasite prevalence may drive the biotic impoverishment of new england (Usa) bumble bee communities. Insects 12, 1–11. https://doi.org/10.3390/insects12100941

Barribeau, S.M., Schmid-Hempel, P., 2013. Qualitatively different immune response of the bumblebee host, *Bombus terrestris*, to infection by different genotypes of the trypanosome gut parasite, *Crithidia bombi*. Infect. Genet. Evol. 20, 249–256. https://doi.org/10.1016/j.meegid.2013.09.014

Brown, M.J.F., Schmid-Hempel, R., Schmid-Hempel, P., 2003. Strong context-dependent virulence in a host-parasite system: Reconciling genetic evidence with theory. J. Anim. Ecol. 72, 994–1002. https://doi.org/10.1046/J.1365-2656.2003.00770.X

Cilia, G., Flaminio, S., Zavatta, L., Ranalli, R., Quaranta, M., Bortolotti, L., Nanetti, A., 2022. Occurrence of Honey Bee (*Apis mellifera* L.) Pathogens in Wild Pollinators in Northern Italy. Front. Cell. Infect. Microbiol. 12, 814. https://doi.org/10.3389/FCIMB.2022.907489

Cohen, H., Smith, G.P., Sardiñas, H., Zorn, J.F., McFrederick, Q.S., Woodard, S.H., Ponisio, L.C., 2021. Mass-flowering monoculture attracts bees, amplifying parasite prevalence. Proc. R. Soc. B 288. https://doi.org/10.1098/RSPB.2021.1369

Cordes, N., Huang, W.F., Strange, J.P., Cameron, S.A., Griswold, T.L., Lozier, J.D., Solter, L.F., 2012. Interspecific geographic distribution and variation of the pathogens *Nosema bombi* and *Crithidia* species in United States bumble bee populations. J. Invertebr. Pathol. 109, 209–216. https://doi.org/10.1016/j.jip.2011.11.005

Erler, S., Popp, M., Wolf, S., Lattorff, H.M.G., 2012. Sex, horizontal transmission, and multiple hosts prevent local adaptation of *Crithidia bombi*, a parasite of bumblebees (Bombus spp.). Ecol. Evol. 2, 930–940. https://doi.org/10.1002/ece3.250

Figueroa, L.L., Compton, S., Grab, H., Mcart, S.H., 2021. Functional traits linked to pathogen prevalence in wild bee communities. Sci. Rep. 11, 7529. https://doi.org/10.1038/s41598-021-87103-3

Figueroa, L.L., Grab, H., Ng, W.H., Myers, C.R., Graystock, P., McFrederick, Q.S., McArt, S.H., 2020. Landscape simplification shapes pathogen prevalence in plant-pollinator networks. Ecol. Lett. 23, 1212–1222. https://doi.org/10.1111/ele.13521

Gegear, R.J., Otterstatter, M.C., Thomson, J.D., 2005. Does parasitic infection impair the ability of bumblebees to learn flower-handling techniques? Anim. Behav. 70, 209–215. https://doi.org/10.1016/j.anbehav.2004.09.025

Goulson, D., Whitehorn, P., Fowley, M., 2012. Influence of urbanisation on the prevalence of protozoan parasites of bumblebees. Ecol. Entomol. 37, 83–89. https://doi.org/10.1111/j.1365-2311.2011.01334.x

Graystock, P., Ng, W.H., Parks, K., Tripodi, A.D., Muñiz, P.A., Fersch, A.A., Myers, C.R., Mcfrederick, Q.S., Mcart, S.H., 2020. Dominant bee species and floral abundance drive parasite temporal dynamics in plant-pollinator communities HHS Public Access. Nat Ecol Evol 4, 1358–1367. https://doi.org/10.1038/s41559-020-1247-x

Korner, P., Schmid-Hempel, P., 2005. Correlates of parasite load in bumblebees in an Alpine habitat. Entomol. Sci. 8, 151–160. https://doi.org/10.1111/j.1479-8298.2005.00113.x

Lim, H.C., Lambrecht, D., Forkner, R.E., Roulston, T., 2023. Minimal sharing of nosematid and trypanosomatid parasites between honey bees and other bees, but extensive sharing of *Crithidia* between bumble and mason bees. J. Invertebr. Pathol. 198, 107933. https://doi.org/10.1016/J.JIP.2023.107933

Malfi, R.L., McFrederick, Q.S., Lozano, G., Irwin, R.E., Adler, L.S., 2023. Sunflower plantings reduce a common gut pathogen and increase queen production in common eastern bumblebee colonies. Proc. R. Soc. B 290. https://doi.org/10.1098/RSPB.2023.0055

Malfi, R.L., Roulston, T.H., 2014. Patterns of parasite infection in bumble bees (*Bombus* spp.) of Northern Virginia. Ecol. Entomol. 39, 17–29. https://doi.org/10.1111/een.12069

Michalczyk, M., Sokół, R., 2022. Detection of *Lotmaria passim* and *Crithidia mellificae* in Selected Bumblebee Species. Pathogens 11. https://doi.org/10.3390/pathogens11091053

Ngor, L., Palmer-Young, E.C., Burciaga Nevarez, R., Russell, K.A., Leger, L., Giacomini, S.J., Pinilla-Gallego, M.S., Irwin, R.E., McFrederick, Q.S., 2020. Cross-infectivity of honey and bumble bee-associated parasites across three bee families. Parasitology 147, 1290–1304. https://doi.org/10.1017/S0031182020001018

Ocepek, M.P., Toplak, I., Zajc, U., Bevk, D., 2021. The Pathogens Spillover and Incidence Correlation in Bumblebees and Honeybees in Slovenia. Pathog. 2021, Vol. 10, Page 884 10, 884. https://doi.org/10.3390/PATHOGENS10070884

Pinilla-Gallego, M.S., Williams, E.E., Davis, A., Fitzgerald, J.L., McArt, S.H., Irwin, R.E., 2020. Within-colonytransmission of microsporidian and trypanosomatid parasites in honey bee and bumble bee colonies. Environ. Entomol. 49, 1393–1401. https://doi.org/10.1093/ee/nvaa112

Plischuk, S., Antúnez, K., Haramboure, M., Minardi, G.M., Lange, C.E., 2017. Long-term prevalence of the protists *Crithidia bombi* and *Apicystis bombi* and detection of the microsporidium *Nosema bombi* in invasive bumble bees. Environ. Microbiol. Rep. 9, 169–173. https://doi.org/10.1111/1758-2229.12520

Plischuk, S., Fernández de Landa, G., Revainera, P., Quintana, S., Pocco, M.E., Cigliano, M.M., Lange, C.E., 2021. Parasites and pathogens associated with native bumble bees (Hymenoptera: Apidae: Bombus spp.) from highlands in Bolivia and Peru. Stud. Neotrop. Fauna Environ. 56, 93–98. https://doi.org/10.1080/01650521.2020.1743551

Plischuk, S., Lange, C.E., 2009. Invasive *Bombus terrestris* (Hymenoptera: Apidae) parasitized by a flagellate (Euglenozoa: Kinetoplastea) and a neogregarine (Apicomplexa: Neogregarinorida). J. Invertebr. Pathol. 102, 263–265. https://doi.org/10.1016/j.jip.2009.08.005

Popp, M., Erler, S., Lattorff, H.M.G., 2012. Seasonal variability of prevalence and occurrence of multiple infections shape the population structure of *Crithidia bombi*, an intestinal parasite of bumblebees (Bombus spp.). Microbiologyopen 1, 362–372. https://doi.org/10.1002/mbo3.35

Ruiz-González, M.X., Brown, M.J.F., 2006. Honey bee and bumblebee trypanosomatids: Specificity and potential for transmission. Ecol. Entomol. 31, 616–622. https://doi.org/10.1111/j.1365-2311.2006.00823.x

Schmid-Hempel, P., Schmid-Hempel, R., 1993. Transmission of a pathogen in *Bombus terrestris*, with a note on division of labour in social insects. Behav. Ecol. Sociobiol. 33, 319–327. https://doi.org/10.1007/BF00172930

Shykoff, J.A., Schmid-Hempel, P., 1991. Genetic relatedness and eusociality: parasite-mediated selection on the genetic composition of groups. Behav. Ecol. Sociobiol. 28, 371–376. https://doi.org/10.1007/BF00164387

Theodorou, P., Radzevičiūtė, R., Settele, J., Schweiger, O., Murray, T.E., Paxton, R.J., 2016. Pollination services enhanced with urbanization despite increasing pollinator parasitism. Proc. R. Soc. B Biol. Sci. 283. https://doi.org/10.1098/rspb.2016.0561

Tiritelli, R., Flaminio, S., Zavatta, L., Ranalli, R., Giovanetti, M., Grasso, D.A., Leonardi, S., Bonforte, M., Boni, C.B., Cargnus, E., Catania, R., Coppola, F., Santo, M. Di, Pusceddu, M., Quaranta, M., Bortolotti, L., Nanetti, A., Cilia, G., 2024. Ecological and social factors influence interspecific pathogens occurrence among bees. Sci. Rep. 1–16. https://doi.org/10.1038/s41598-024-55718-x

Tommasi, N., Colombo, B., Pioltelli, E., Biella, P., Casiraghi, M., Galimberti, A., 2023. Urban habitat fragmentation and floral resources shape the occurrence of gut parasites in two bumblebee species. Ecol. Evol. 13. https://doi.org/10.1002/ECE3.10299

Tripodi, A.D., Cibils-Stewart, X., McCornack, B.P., Szalanski, A.L., 2014. *Nosema bombi* (Microsporidia: Nosematidae) and Trypanosomatid Prevalence in Spring Bumble Bee Queens (Hymenoptera: Apidae: Bombus) in Kansas. https://doi.org/10.2317/JKES130730.1 87, 225–233. https://doi.org/10.2317/JKES130730.1

Tripodi, A.D., Szalanski, A.L., Strange, J.P., 2018. Novel multiplex PCR reveals multiple trypanosomatid species infecting North American bumble bees (Hymenoptera: Apidae: Bombus). J. Invertebr. Pathol. 153, 147–155. https://doi.org/10.1016/J.JIP.2018.03.009

Vavilova, V.Y., Konopatskaia, I., Luzyanin, S.L., Woyciechowski, M., Blinov, A.G., 2017. Parasites of the genus *Nosema*, *Crithidia* and *Lotmaria* in the honeybee and bumblebee populations: A case study in India. Vavilovskii Zhurnal Genet. Selektsii 21, 943–951. https://doi.org/10.18699/VJ17.317

Votavová, A., Trněný, O., Staveníková, J., Dybová, M., Brus, J., Komzáková, O., 2022. Prevalence and Distribution of Three Bumblebee Pathogens from the Czech Republic. Insects 13. https://doi.org/10.3390/INSECTS13121121/S1
