# Supplementary material for: The trypanosomatid (Kinetoplastida: Trypanosomatidae) parasites in bees: A review on their environmental circulation, impacts and implications
Source: Curr Res Insect Sci. 2025 Jan 21;7:100106. doi: 10.1016/j.cris.2025.100106 (PMC11803887; doi:10.1016/j.cris.2025.100106)
Supplement: Supplementary file 2 [file mmc2.docx]

**Table S2.** Detection of various wild bee species infected with trypanosomatids across different countries.

| **Country** | **Trypanosomatids** | **Wild bee species** | **References** |
| --- | --- | --- | --- |
| AR | *C. bombi* | *Eucera fervens, Lasioglossum* spp., *Xylocopa augusti* | (Fernandez De Landa et al., 2023) |
| BE | *C. bombi* | *Andrena vaga, Osmia bicornis* | (Ravoet et al., 2014) |
| IT | *L. passim* | *Andrena ferrigineus, Andrena labiata, A. ventricosa, A. flavipes, A. humilis, A. miegella, A. pastellensis, Anthophora dispar, A. plumipes, Chelostoma rapunculi, Ch. florisomme, Halictus simplex, Hylaeus clypearis, E. clypeata, E. eucneumidea, E. nigrescens, E. nigrifacies, L. malachurum, L. marginatum, L. politum, L. xanthopus, M. pilidens, O. bicornis, O. notata, Seladonia gemmea* | (Cilia et al., 2022; Tiritelli et al., 2024) |
|  | *C. bombi* | *A. flavipes, A. impuctata, Anthidium florentinum, Ceratina cucurbitina, C. cyanea, E. eucnemidea, E. nigrifacies, Heriades crenulate, Hylaeus* spp., *L. marginatum, L. glabriusculum, L. leucozonium, L. villosulum, O. latrillei, Pseudanthidium nanum, S. subaurata* |  |
| CH | *C. mellificae** | *O. cornuta* | (Strobl et al., 2019) |
| US | Trypanosomatidae spp. | *Agapostemon viriscens, Anthophora teminalis, Augocholorella aurata, C. calcarata, C. dupla, C. mikmaqi, H. confuses, H. ligatus, Hy. affinis, Hy. modestus, L. pilosum, L. viridatum, Megachile* spp., *Melissodes agilis, Mel. trinodis, X. virginica* | (Figueroa et al., 2020, 2021) |
|  | *Crithidia* spp. | *H. tripartitus, H. ligatus, Hylaeus* spp., *L. tegulariforme, Mel. Agilis* | (Cohen et al., 2021) |
|  | *C. bombi* | *Au. Aurata, Au. pura, C. calcarata, C. mikmaqi, Colletes simulans, H. ligatus, L. zonolum, M. rotundata, M. montivaga, O. lignaria, O. taurus, O. cornifrons, X. virginica* | (Figueroa et al., 2020; Graystock et al., 2020; Lim et al., 2023; Ngor et al., 2020) |
|  | *C. expoeki* | *H. tripartitus, H. ligatus, L. zonulum, L. tegulariforme, L. diversopunctatum, L. incompletum, Mel. agilis, Mel. lupina, Svastra obliqua, X. virginica* | (Cohen et al., 2021; Graystock et al., 2020) |
|  | *C. mellificae* | *H. ligatus, O. lignaria* | (Ngor et al., 2020) |

Note: *: artificial infection; AR: Argentina; BE: Belgium; CH: Switzerland; IT: Italy; US: USA.

**References**

Cilia, G., Flaminio, S., Zavatta, L., Ranalli, R., Quaranta, M., Bortolotti, L., Nanetti, A., 2022. Occurrence of Honey Bee (*Apis mellifera* L.) Pathogens in Wild Pollinators in Northern Italy. Front. Cell. Infect. Microbiol. 12, 814. https://doi.org/10.3389/FCIMB.2022.907489

Cohen, H., Smith, G.P., Sardiñas, H., Zorn, J.F., McFrederick, Q.S., Woodard, S.H., Ponisio, L.C., 2021. Mass-flowering monoculture attracts bees, amplifying parasite prevalence. Proc. R. Soc. B 288. https://doi.org/10.1098/RSPB.2021.1369

Fernandez De Landa, G., Alberoni, D., Baffoni, L., Fernandez De Landa, M., Revainera, P.D., Porrini, L.P., Brasesco, C., Quintana, S., Zumpano, F., Eguaras, M.J., Maggi, M.D., Di Gioia, D., 2023. The gut microbiome of solitary bees is mainly affected by pathogen assemblage and partially by land use. Environ. Microbiome 18, 1–17. https://doi.org/10.1186/s40793-023-00494-w

Figueroa, L.L., Grab, H., Ng, W.H., Myers, C.R., Graystock, P., McFrederick, Q.S., McArt, S.H., 2020. Landscape simplification shapes pathogen prevalence in plant-pollinator networks. Ecol. Lett. 23, 1212–1222. https://doi.org/10.1111/ele.13521

Figueroa, L.L., Grincavitch, C., McArt, S.H., 2021. *Crithidia bombi* can infect two solitary bee species while host survivorship depends on diet. Parasitology 148, 435–442. https://doi.org/10.1017/S0031182020002218

Graystock, P., Ng, W.H., Parks, K., Tripodi, A.D., Muñiz, P.A., Fersch, A.A., Myers, C.R., Mcfrederick, Q.S., Mcart, S.H., 2020. Dominant bee species and floral abundance drive parasite temporal dynamics in plant-pollinator communities HHS Public Access. Nat Ecol Evol 4, 1358–1367. https://doi.org/10.1038/s41559-020-1247-x

Lim, H.C., Lambrecht, D., Forkner, R.E., Roulston, T., 2023. Minimal sharing of nosematid and trypanosomatid parasites between honey bees and other bees, but extensive sharing of *Crithidia* between bumble and mason bees. J. Invertebr. Pathol. 198, 107933. https://doi.org/10.1016/J.JIP.2023.107933

Ngor, L., Palmer-Young, E.C., Burciaga Nevarez, R., Russell, K.A., Leger, L., Giacomini, S.J., Pinilla-Gallego, M.S., Irwin, R.E., McFrederick, Q.S., 2020. Cross-infectivity of honey and bumble bee-associated parasites across three bee families. Parasitology 147, 1290–1304. https://doi.org/10.1017/S0031182020001018

Ravoet, J., De Smet, L., Meeus, I., Smagghe, G., Wenseleers, T., de Graaf, D.C., 2014. Widespread occurrence of honey bee pathogens in solitary bees. J. Invertebr. Pathol. 122, 55–58. https://doi.org/10.1016/j.jip.2014.08.007

Strobl, V., Yañez, O., Straub, L., Albrecht, M., Neumann, P., 2019. Trypanosomatid parasites infecting managed honeybees and wild solitary bees. Int. J. Parasitol. 49, 605–613. https://doi.org/10.1016/J.IJPARA.2019.03.006

Tiritelli, R., Flaminio, S., Zavatta, L., Ranalli, R., Giovanetti, M., Grasso, D.A., Leonardi, S., Bonforte, M., Boni, C.B., Cargnus, E., Catania, R., Coppola, F., Santo, M. Di, Pusceddu, M., Quaranta, M., Bortolotti, L., Nanetti, A., Cilia, G., 2024. Ecological and social factors influence interspecific pathogens occurrence among bees. Sci. Rep. 1–16. https://doi.org/10.1038/s41598-024-55718-x
